# 538 Demographic and Injury Differences Between Children with Burn Injury from the United States and Mexico

**DOI:** 10.1093/jbcr/irad045.135

**Published:** 2023-05-15

**Authors:** Victoria Miles, oscar Suman-Vejas, Colleen Ryan, Barclay Stewart, Gretchen Carrougher, Caitlin Orton, Karen Kowalske, Bhaskar Thakur, Kyra Jeanine Solis, Sujata Dalal

**Affiliations:** University of Tennessee College of Medicine Chattanooga, Chattanooga, Tennessee; The University of Texas Medical Branch, Galveston, Texas; Massachusetts General Hospital, Boston, Massachusetts; University of Washington, Seattle, Washington; University of Washington, Seattle, Washington; University of Washington, Seattle, Washington; UT Southwestern Medical Center, Dallas, Dallas, Texas; UT Southwestern Medical Center, Dallas, Texas; UT Southwestern Medical Center, Dallas, Texas; Texas A&M School of Medicine, Dallas, Texas; University of Tennessee College of Medicine Chattanooga, Chattanooga, Tennessee; The University of Texas Medical Branch, Galveston, Texas; Massachusetts General Hospital, Boston, Massachusetts; University of Washington, Seattle, Washington; University of Washington, Seattle, Washington; University of Washington, Seattle, Washington; UT Southwestern Medical Center, Dallas, Dallas, Texas; UT Southwestern Medical Center, Dallas, Texas; UT Southwestern Medical Center, Dallas, Texas; Texas A&M School of Medicine, Dallas, Texas; University of Tennessee College of Medicine Chattanooga, Chattanooga, Tennessee; The University of Texas Medical Branch, Galveston, Texas; Massachusetts General Hospital, Boston, Massachusetts; University of Washington, Seattle, Washington; University of Washington, Seattle, Washington; University of Washington, Seattle, Washington; UT Southwestern Medical Center, Dallas, Dallas, Texas; UT Southwestern Medical Center, Dallas, Texas; UT Southwestern Medical Center, Dallas, Texas; Texas A&M School of Medicine, Dallas, Texas; University of Tennessee College of Medicine Chattanooga, Chattanooga, Tennessee; The University of Texas Medical Branch, Galveston, Texas; Massachusetts General Hospital, Boston, Massachusetts; University of Washington, Seattle, Washington; University of Washington, Seattle, Washington; University of Washington, Seattle, Washington; UT Southwestern Medical Center, Dallas, Dallas, Texas; UT Southwestern Medical Center, Dallas, Texas; UT Southwestern Medical Center, Dallas, Texas; Texas A&M School of Medicine, Dallas, Texas; University of Tennessee College of Medicine Chattanooga, Chattanooga, Tennessee; The University of Texas Medical Branch, Galveston, Texas; Massachusetts General Hospital, Boston, Massachusetts; University of Washington, Seattle, Washington; University of Washington, Seattle, Washington; University of Washington, Seattle, Washington; UT Southwestern Medical Center, Dallas, Dallas, Texas; UT Southwestern Medical Center, Dallas, Texas; UT Southwestern Medical Center, Dallas, Texas; Texas A&M School of Medicine, Dallas, Texas; University of Tennessee College of Medicine Chattanooga, Chattanooga, Tennessee; The University of Texas Medical Branch, Galveston, Texas; Massachusetts General Hospital, Boston, Massachusetts; University of Washington, Seattle, Washington; University of Washington, Seattle, Washington; University of Washington, Seattle, Washington; UT Southwestern Medical Center, Dallas, Dallas, Texas; UT Southwestern Medical Center, Dallas, Texas; UT Southwestern Medical Center, Dallas, Texas; Texas A&M School of Medicine, Dallas, Texas; University of Tennessee College of Medicine Chattanooga, Chattanooga, Tennessee; The University of Texas Medical Branch, Galveston, Texas; Massachusetts General Hospital, Boston, Massachusetts; University of Washington, Seattle, Washington; University of Washington, Seattle, Washington; University of Washington, Seattle, Washington; UT Southwestern Medical Center, Dallas, Dallas, Texas; UT Southwestern Medical Center, Dallas, Texas; UT Southwestern Medical Center, Dallas, Texas; Texas A&M School of Medicine, Dallas, Texas; University of Tennessee College of Medicine Chattanooga, Chattanooga, Tennessee; The University of Texas Medical Branch, Galveston, Texas; Massachusetts General Hospital, Boston, Massachusetts; University of Washington, Seattle, Washington; University of Washington, Seattle, Washington; University of Washington, Seattle, Washington; UT Southwestern Medical Center, Dallas, Dallas, Texas; UT Southwestern Medical Center, Dallas, Texas; UT Southwestern Medical Center, Dallas, Texas; Texas A&M School of Medicine, Dallas, Texas; University of Tennessee College of Medicine Chattanooga, Chattanooga, Tennessee; The University of Texas Medical Branch, Galveston, Texas; Massachusetts General Hospital, Boston, Massachusetts; University of Washington, Seattle, Washington; University of Washington, Seattle, Washington; University of Washington, Seattle, Washington; UT Southwestern Medical Center, Dallas, Dallas, Texas; UT Southwestern Medical Center, Dallas, Texas; UT Southwestern Medical Center, Dallas, Texas; Texas A&M School of Medicine, Dallas, Texas

## Abstract

**Introduction:**

A large burn data set is being used to evaluate outcomes of pediatric burn survivors, but questions remain whether this data set is generalizable to the typical population of US burn centers as only 62% of enrollees are from the US. A better understanding of similarities and differences within this data set will help in the applicability of findings from it.

**Methods:**

This is a retrospective cohort study using data from children enrolled in a burn database. Data from 2043 children admitted for burn injury from 1994 to 2020 were analyzed. Data were stratified into children who were US residents and Mexico residents at the time of injury, all treated in US hospitals, and grouped by age into “< 5 years”, “5-10 years” and “ >10 years”. Sex, race, ethnicity, injury etiology, total body surface area injured (TBSA), and circumstances of injury were compared. Fisher’s exact test and Kruskal–Wallis one-way analysis of variance were used to test for differences in categorical and continuous variables, respectively.

**Results:**

Both US and Mexico populations showed increased incidence among males as they aged. In the US population, as expected the most frequent cause of burns in patients < 5 years was scald. In the Mexico population, fire/flame burns were more frequent in all ages and electric burns were more common among children > 5 years. In both populations, indoor injuries were more common among children < 5 years and outdoor injuries were more common as children aged. Children from Mexico had larger burns and longer lengths of hospital stay than US children across all ages.

**Conclusions:**

Children from Mexico who were referred to the US for burn care were similar in demographic patterns to US children. While differences were seen in extent and mechanism, the difference is likely influenced by larger, more severe burns being transferred to the US for care, while low severity burns were treated locally. This suggests that children from Mexico in the database are not representative of the pediatric burn injury population of the US or Mexico more broadly. When studying datasets from US hospitals containing non-US residents, it is recommended that these populations be separately analyzed, otherwise, findings cannot be generalized to the US pediatric burn injury population.

**Applicability of Research to Practice:**

In addition to identifying prevention needs, differences among US and Mexico resident children cared for in the US limit the utility of analyzing this population in the burn dataset.

